# Cefazolin: A Rare Etiology of Induced Coagulopathy

**DOI:** 10.7759/cureus.101938

**Published:** 2026-01-20

**Authors:** Ahmed Ismail, Anam Habib, Cayleigh Blumrick

**Affiliations:** 1 Department of Medicine, Eastern Virginia Medical School at Old Dominion University, Norfolk, USA; 2 Division of Infectious Diseases, Department of Medicine, Eastern Virginia Medical School at Old Dominion University, Norfolk, USA

**Keywords:** aptt, cefazolin, chronic kidney disease, hypoprothrombinemia, infective endocarditis, inr

## Abstract

Cefazolin, a first-generation cephalosporin, is commonly used to treat methicillin-susceptible *Staphylococcus aureus* (MSSA) infections. Although generally well tolerated, coagulation abnormalities have rarely been reported during therapy. We describe the case of an 81-year-old man treated with prolonged cefazolin for MSSA endocarditis who developed severe coagulopathy characterized by marked elevation of both the international normalized ratio (INR) and activated partial thromboplastin time (aPTT). The abnormalities occurred after initiation of cefazolin and worsened despite cessation of other anticoagulants. Following discontinuation of cefazolin and administration of vitamin K, coagulation parameters rapidly improved and subsequently normalized. This case highlights a rare but clinically significant adverse effect of cefazolin and underscores the importance of close coagulation monitoring during prolonged therapy, particularly in high-risk patient populations.

## Introduction

Normal hemostasis depends on a tightly regulated coagulation cascade involving vitamin K-dependent clotting factors, adequate hepatic synthesis, and intact nutritional status. Disruption of any of these components, such as vitamin K deficiency, renal dysfunction, malnutrition, liver disease, or drug-drug interactions, can result in clinically significant coagulopathy. These vulnerabilities are particularly relevant in elderly patients requiring prolonged antimicrobial therapy, which may disrupt coagulation homeostasis.

Cephalosporins are widely prescribed β-lactam antibiotics due to their favorable safety profile and broad-spectrum coverage. However, certain cephalosporins, particularly those containing an N-methylthiotetrazole (NMTT) side chain, have been implicated in coagulation abnormalities through disruption of vitamin K metabolism and inhibition of γ-carboxylation of clotting factors II, VII, IX, and X (e.g., cefotetan, cefoperazone, and cefamandole) [1]. Coagulopathy and clinically significant bleeding are well-documented adverse effects of these agents. Still, they are exceedingly rare with cefazolin, a first-generation cephalosporin that lacks the NMTT but has a methyl-thiadiazole (MTD) thiol side chain [2]. Despite its general safety, isolated reports suggest that cefazolin can precipitate hypoprothrombinemia, especially in patients with underlying risk factors such as renal impairment, malnutrition, prolonged therapy, or concurrent use of other agents that interfere with vitamin K homeostasis [3,4]. Clinicians may overlook this complication due to its rarity, leading to delays in recognition and treatment. We present a rare case of cefazolin-induced hypoprothrombinemia manifesting as a simultaneous and progressive increase in both international normalized ratio (INR) and activated partial thromboplastin time (aPTT) in a patient with infective endocarditis (IE) and chronic kidney disease (CKD), requiring long-term treatment of cefazolin. This case emphasizes the importance of early recognition, risk stratification, and routine monitoring of coagulation parameters in high-risk populations. This case highlights that even antibiotics considered safe can lead to potentially life-threatening adverse effects.

## Case presentation

Our case is an 81-year-old man with a past medical history of chronic obstructive pulmonary disease, coronary artery disease, atrial fibrillation on warfarin, automatic implantable cardioverter-defibrillator (AICD), heart failure with preserved ejection fraction, diabetes mellitus, hypertension, hyperlipidemia, deep venous thrombosis, obstructive sleep apnea with poor compliance to therapy, and stage 4 CKD. He presented to the emergency department with progressive shortness of breath and cough. On arrival, he was hemodynamically stable but hypoxemic with oxygen saturations in the 70s, requiring bilevel positive airway pressure (BiPAP) support. Physical examination revealed bilateral crackles on lung auscultation and lower extremity edema consistent with volume overload. Cardiac examination demonstrated an irregular rate and rhythm without audible murmurs. There were no peripheral stigmata of infective endocarditis, including Janeway lesions, Osler nodes, splinter hemorrhages, or conjunctival petechiae.

**Figure 1:**
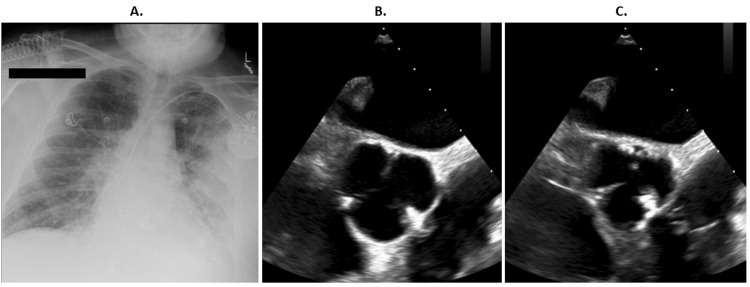
Representative Imaging of Chest X-Ray and Transesophageal Echocardiography A: Chest radiograph demonstrating patchy left-sided airspace opacities consistent with pneumonia. Pulmonary edema was also a possibility, but less likely. B and C: Transesophageal echocardiography images demonstrating a small fibrinous echo density on the left coronary cusp of the aortic valve, which could not exclude infective endocarditis.

Initial pertinent laboratory studies are summarized in Table 1. Chest radiography (X-ray) revealed new patchy airspace opacities throughout the left lung consistent with pneumonia (Figure 1). He was admitted with diagnoses of acute kidney injury (AKI) on CKD, acute exacerbation of heart failure, acute on chronic hypoxemic respiratory failure, and pneumonia. Management included BiPAP, intravenous furosemide, metoprolol, and empiric antibiotic therapy with linezolid and cefepime.

**Table 1:** Initial Laboratory Results on Presentation WBC: white blood cell, RBC: red blood cell, HGB: hemoglobin, HCT: hematocrit, MCV: mean corpuscular volume, MCH: mean corpuscular hemoglobin, MCHC: mean corpuscular hemoglobin concentration, RDW: red cell distribution width, MPV: mean platelet volume, PT: prothrombin time, INR: international normalized ratio, BUN: blood urea nitrogen, NT-proBNP: N-terminal pro-B-type natriuretic peptide, A/G ratio: albumin/globulin ratio, SGOT: serum glutamic-oxaloacetic transaminase, AST: aspartate aminotransferase, SGPT: serum glutamic-pyruvic transaminase, ALT: alanine aminotransferase, BiPAP: bilevel positive airway pressure, PCO2: partial pressure of carbon dioxide, PO2: partial pressure of oxygen

Laboratory test	Reference range	Result
WBC	4-11 K/uL	15.1 K/uL
RBC	3.80-5.80 M/uL	3.41 M/uL
HGB	12.6-17.1 g/dL	10.4 g/dL
HCT	37.8%- 52.2%	32.1%
MCV	80-95 fL	94 fL
MCH	26-34 pg	31 pg
MCHC	31-36 g/dL	32 g/dL
RDW	10.0%-15.5%	13.8%
Platelet	140-440 K/uL	187 K/uL
MPV	9-13 fL	10.2 fL
Segmented neutrophils	40%-75%	92% (high)
Lymphocytes	20%-45%	2% (low)
Monocytes	3%-12%	5%
Eosinophils	0%-6%	0%
Basophils	0%-2%	0%
Absolute neutrophils	1.8-7.7 K/uL	14 K/uL (high)
Absolute lymphocytes	1.0-4.8 K/uL	0.3 K/uL (low)
Absolute monocytes	0.1-1.0 K/uL	0.8 K/uL
Absolute eosinophils	0.0-0.5 K/uL	0 K/uL
Absolute basophils	0.0-0.2 K/uL	0 K/uL
PT	9-13 seconds	22 seconds
INR	0.93-1.29	2.2
Creatinine	0.6-1.2 mg/dL	4.2 mg/dL
BUN	7-20 mg/dL	84 mg/dL
Glucose	70-100 mg/dL	265 mg/dL
NT-proBNP	<125 pg/mL (<75 years), <450 pg/mL (>75 years)	19,627 pg/mL
Lactic acid	<0.05 ng/mL (normal), 0.05-0.5 ng/mL (low risk), >0.5 ng/mL (bacterial infection)	2.8 ng/mL
Procal	0.5-2.0 mmol/L	0.6 0 mmol/L
Albumin	3.5-5.0 g/dL	3.5 g/dL
Total protein	6.2-8.1 g/dL	6.6 g/dL
Globulin	2-4 g/dL	3.5 g/dL
A/G ratio	1.1-2.6	3.1
Bilirubin (total)	0.2-1.2 mg/dL	0.9 mg/dL
Bilirubin (direct)	0.0-0.3 mg/dL	0.3 mg/dL
SGOT (AST)	10-37 U/L	54 U/L
Alkaline phosphatase	40-125 U/L	91 U/L
SGPT (ALT)	5-40 U/L	37 U/L
Arterial blood gas after BiPAP (on presentation)		
PH	7.350-7.450	7.35
PCO2	34-45 mmHg	34 mmHg
PO2	80-100 mmHg	71 mmHg
Bicarbonate	22-26 mmol/L	18.9 mmol/L
%O2 saturation arterial	96%-100%	94%

Blood cultures obtained on admission grew methicillin-sensitive *Staphylococcus aureus* (MSSA) in both aerobic and anaerobic bottles (Table 2). The suspected source was chronic lower extremity wounds. Transthoracic echocardiography (TTE) showed an ejection fraction of 55% and was unrevealing for any abnormalities. However, transesophageal echocardiography (TEE) demonstrated a tricuspid aortic valve with a small fibrinous echo density on the left coronary cusp, possibly fibrinous stranding, but could not exclude endocarditis (Figure 1). Notably, the AICD leads showed no vegetation.

**Table 2:** Staphylococcus aureus Sensitivity Results MIC: minimum inhibitory concentration

Antibiotic	MIC	Sensitivity
Cefazolin (deduced)	-	Sensitive
Clindamycin	0.25	Sensitive
Gentamicin	≤0.5	Sensitive
Oxacillin	0.5	Sensitive
Rifampin	≤0.5	Sensitive
Tetracycline	≤1	Sensitive

The diagnosis of infective endocarditis was supported by modified Duke criteria, including two major criteria: persistent MSSA bacteremia and echocardiographic findings suggestive of valvular involvement. Therefore, the overall clinical presentation warranted treatment as definite infective endocarditis. Although the AICD leads showed no vegetation, the device was removed and replaced with a leadless system due to the clinical context. Antibiotics were narrowed to renally dosed cefazolin (2 gm every 12 hours, instead of the full dose of 2 gm every eight hours), with a planned six-week course.

**Figure 2:**
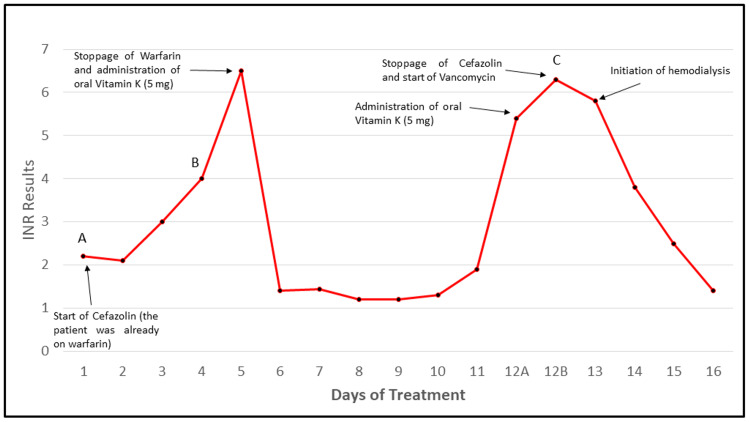
Temporal Trends in INR During Cefazolin Therapy INR values are plotted over the course of treatment. Key clinical interventions are annotated, including discontinuation of warfarin, administration of vitamin K, initiation of hemodialysis, and cessation of cefazolin. aPTT measurements were obtained at three time points in A, B, and C (31 seconds at cefazolin initiation, 50 seconds during initial INR rise, and 53 seconds at peak INR, respectively), demonstrating a parallel elevation with INR. INR: international normalized ratio, aPTT: activated partial thromboplastin time

Five days into cefazolin therapy, the patient’s INR rose sharply to 6.5. Warfarin was discontinued (given a prior history of warfarin toxicity), and oral vitamin K (5 mg) was administered, resulting in a normalization of INR to 1.2 over three days. However, after six days of INR normalization, it began to increase again to a peak of 6.3 (day 12 of cefazolin therapy) (Figure 2) despite cessation of warfarin. Importantly, during this time, the patient’s renal function continued to deteriorate (creatinine: 8.2 mg/dL from 4.2 mg/dL, BUN: 120 mg/dL from 82 mg/dL), prompting getting a dialysis catheter and initiation of hemodialysis. Further workup (Table 3) included a coagulation panel, which revealed markedly prolonged prothrombin time (58 seconds), elevated INR (5.8), prolonged aPTT (58 seconds), mildly elevated D-dimer (1.27 mg/L FEU), and elevated fibrinogen (634 mg/dL). Platelet counts remained within normal limits, and there was no clinical or laboratory evidence of hemolysis, making disseminated intravascular coagulation unlikely. The patient was not receiving heparin and had not yet started hemodialysis at that time, excluding heparin-induced aPTT prolongation and dialysis-associated anticoagulation. Liver function tests were within normal limits, arguing against hepatic synthetic dysfunction as the cause of coagulopathy.

**Table 3:** Pertinent Laboratory Results After Initiation of Cefazolin BUN: blood urea nitrogen, A/G ratio: albumin/globulin ratio, SGOT: serum glutamic-oxaloacetic transaminase, AST: aspartate aminotransferase, SGPT: serum glutamic-pyruvic transaminase, ALT: alanine aminotransferase, LDH: lactate dehydrogenase, INR: international normalized ratio, PT: prothrombin time, activated partial thromboplastin time

Laboratory test	Reference range	Result
Creatinine	0.6-1.2 mg/dL	8.2 mg/dL
BUN	7-20 mg/dL	120 mg/dL
Platelet	140-440 K/uL	147 K/uL
Albumin	3.5-5.0 g/dL	2.5 g/dL
Total protein	6.2-8.1 g/dL	6 g/dL
Globulin	2-4 g/dL	3.5 g/dL
A/G ratio	1.1-2.6	0.7
Bilirubin (total)	0.2-1.2 mg/dL	0.3 mg/dL
Bilirubin (direct)	0.0-0.3 mg/dL	<0.2 mg/dL
SGOT (AST)	10-37 U/L	17 U/L
Alkaline phosphatase	40-125 U/L	63 U/L
SGPT (ALT)	5-40 U/L	<5 U/L
LDH	98-192 U/L	283 U/L
Haptoglobin	30-200 mg/dL	268 mg/dL
Coagulation studies		
D-dimer	0.00-1.12 mg/L FEU	1.27 mg/L FEU
INR	0.93-1.29	5.8
PT	9-13 seconds	58 seconds
aPTT	22-36 seconds	58 seconds
Fibrinogen	200-425 mg/dL	634 mg/dL

Given the temporal association, normal hepatic function, and absence of disseminated intravascular coagulation (DIC) features, cefazolin-induced coagulopathy was suspected, likely exacerbated by impaired renal clearance. Cefazolin was discontinued and replaced with vancomycin, which was administered during dialysis sessions due to its predictable clearance in end-stage renal dysfunction. Oral vitamin K (5 mg) was administered, resulting in a rapid improvement in coagulation parameters (INR of 3.8 within one day and nearly normalized by four days at 1.4). The patient remained stable and experienced no bleeding episodes during the remainder of the hospitalization. He was discharged in stable condition, on apixaban instead of warfarin, with appropriate follow-up.

## Discussion

Cefazolin, a first-generation cephalosporin, is one of the most frequently prescribed antibiotics worldwide. It is routinely employed for surgical prophylaxis [5]. It is also considered a first-line option for treating methicillin-sensitive *Staphylococcus aureus* infections due to its proven efficacy, affordability, and favorable safety profile [6]. Although generally well tolerated, cefazolin has been associated with the uncommon but clinically significant complication of hypoprothrombinemia, which may result in life-threatening bleeding events [3]. Coagulopathy has been more frequently associated with cephalosporins containing the N-methylthiotetrazole (NMTT) group, which interferes with the vitamin K-dependent γ-carboxylation of glutamic acid residues, leading to impaired hemostasis [7]. Although cefazolin lacks NMTT, it has a similar thiol side chain, MTD, implicated in similar effects [2].

In 2017, a series of five patients in France developed severe bleeding events linked to hypoprothrombinemia and elevated INR while receiving high-dose cefazolin therapy [8]. Subsequently, a 2018 study demonstrated that cefazolin therapy was associated with significant prolongation of aPTT (>25 seconds), without a corresponding increase in median INR, with older age and reduced renal function identified as the primary risk factors [7]. Their findings showed clinically significant bleeding occurred in 5% of patients, highlighting the need for careful monitoring in high-risk populations, particularly during prolonged therapy [7].

Several predisposing factors have been identified that increase the risk of cefazolin-induced coagulopathy. CKD or renal impairment, along with old age, serves as one of the most significant risk factors, likely due to impaired renal clearance and drug accumulation [7]. Poor nutritional status may further increase susceptibility by reducing vitamin K reserves [2]. Prolonged cefazolin therapy has also been suggested as a risk factor [8]. In addition, co-administration of agents that interfere with vitamin K homeostasis, such as rifampin, may exacerbate coagulopathy, which is well-documented in the literature [4].

Our case illustrates an 81-year-old man with AKI on CKD, with no hepatic dysfunction or DIC, requiring long-term treatment with cefazolin secondary to MSSA endocarditis. Such a combination of risk factors likely predisposed the patient to cefazolin-induced coagulopathy. Impaired renal clearance, in addition to long-term cefazolin use, may have led to accumulation of cefazolin and its metabolites, potentially enhancing its effects on coagulation. Additionally, the initial presentation with acute exacerbation of heart failure and volume overload might have caused a component of acute congestive hepatopathy (Table 1), which might have further worsened coagulopathy (only during the initial peak of INR in Figure 2). Interestingly, unlike prior reports, our case demonstrates simultaneous elevation of both INR and aPTT, underscoring the importance of monitoring both parameters in high-risk patients. A limitation in our case report is that aPTT was not monitored daily. However, a marked spike in aPTT was observed after cefazolin initiation, coinciding with the INR spike (Figure 2).

Management of cefazolin-induced coagulopathy consists of prompt discontinuation of the offending agent, administration of vitamin K (parenteral or oral, depending on the severity), and initiation of an alternative antibiotic that lacks the implicated side chain structure. Previous studies have shown that vitamin K therapy is effective in reversing such coagulopathy [1]. In clinical practice, switching to agents outside the cephalosporin class, such as vancomycin or other beta-lactams without the implicated side chain, is recommended to prevent recurrence [9]. In our case, cefazolin was switched to vancomycin, with re-administration of vitamin K. This resulted in rapid correction of coagulation abnormalities and normalization of the INR within three days, consistent with previously reported favorable outcomes following timely intervention [3,8].

Despite these data in the literature, there remain no clinical trials or large-scale cohort studies specifically evaluating the incidence of coagulopathy or hemorrhagic complications associated with cefazolin use, to the best of our knowledge. It would be beneficial to explore such entities further.

## Conclusions

Our case report highlights the importance of early recognition, risk stratification, and routine monitoring of coagulation parameters, including INR/aPTT, in high-risk patients undergoing prolonged cefazolin therapy. Unlike previously published reports that predominantly emphasize INR elevation, our case demonstrated simultaneous and progressive prolongation of both INR and aPTT, suggesting a broader disturbance of the coagulation cascade. Clinicians may overlook this rare complication due to its infrequency, potentially leading to life-threatening bleeding and delays in recognition and treatment. Prompt action, including discontinuation of cefazolin and vitamin K supplementation, can effectively mitigate the risk of bleeding and prevent severe hemorrhagic events.
